# Women's colposcopy experience and preferences: a mixed methods study

**DOI:** 10.1186/1472-6874-8-2

**Published:** 2008-01-14

**Authors:** Dawn R Swancutt, Sheila M Greenfield, Sue Wilson

**Affiliations:** 1 Department of Primary Care & General Practice, University of Birmingham, Edgbaston, Birmingham, B15 2TT, UK

## Abstract

**Background:**

The colposcopy service is a key component in the UK Cervical Screening Programme. Over 120,000 women are referred to the service annually, however up to 25% of women fail to attend their appointment. Little is known about patients' preferences for colposcopic investigation and treatment. This study aims to investigate women's experience of colposcopy, to identify patients' preferences for aspects of appointments within the colposcopy service, and to make suggestions for service improvement.

**Methods/Design:**

This study has been designed as a two stage, mixed method project. Stage one will involve in-depth interviews with new colposcopy patients to ascertain their experience of colposcopy services. This qualitative stage will generate factors thought to be important by service users in their experience. Stage two will utilise a choice based quantitative technique to identify women's preferences and determine the representativeness of factors generated through the interviews.

The initial stage of in-depth interviews will be conducted with patients who are newly referred to colposcopy clinics to investigate the experience that they have of the referral process and appointment attendance. The outcome of these interviews will be analysed qualitatively using Framework analysis. Factors found to be important in women's experience will be extracted and used to construct a choice based questionnaire.

The discrete choice experiment (questionnaire) will apply a best-worst technique through scenario-based questions to find women's relative preferences for different aspects of the service. It will be offered to women attending follow-up appointments at two colposcopy clinics in the West Midlands. Women will complete the questionnaire whilst they wait for their appointment, or, if they prefer, will take it home to complete in private. Women who do not attend their appointment will be posted the research information and questionnaire. The questionnaire analysis will use a weighted least squares regression technique for each best/worst pair. The accept/reject 'would you attend this appointment' question will be analysed using a random effects logit model.

**Discussion:**

Colposcopy is a common procedure and one that is associated with raised anxiety among women experiencing the service. Little is known about women's experience of the service or their preferences for service delivery. The outcomes of the study will comprise a description of women's experience of colposcopy and establishing their preferences for how aspects of the service should be provided. Women's preferences will be fed back to service providers to enable improvements to the service to be made.

## Background

The cervical screening programme is a key component of UK health policy addressing the primary prevention of cervical cancer. Colposcopy is the accepted procedure for the diagnosis and treatment of abnormal cervical cells. The growing number of abnormal smears referred for colposcopy has increased pressure on services, resulting in prolonged waiting times for patients at such an anxious time [1].

Colposcopy practice has undergone considerable changes over recent years [2]. Nurse colposcopists have emerged during the last decade in response to increased demands for this service. A retrospective analysis of routinely collected data [3] suggested that nurse colposcopists provide a level of service at least as good as that provided by gynaecologists. Pilot work (Unpublished: Lester H, Luesley D. Psychological trauma of the abnormal smear, in a practice and a hospital setting. BSCCP conference, Glasgow, 1997) has suggested that, compared with a doctor led colposcopy service, many women prefer a nurse led service.

Up to 25% of women fail to attend their initial colposcopy appointment [4]. Factors influencing colposcopy attendance include childcare commitments, fear and anxiety, level of understanding about colposcopy, accessibility of information and when staff attitudes were perceived as insensitive [5]. 'No show' behaviour is more likely in women under 30 years of age or pregnant [6]. There is conflicting evidence on the importance of race, follow up letters or grade of lesion [7,8]. A review of methods of reducing non-attendance rates (indicating potential deficiencies in the system of service delivery) suggested the need to tailor any intervention to the population and the type of information to the individual [9]. Identification of inequalities in colposcopy provision (e.g. cultural, age based or socio-economic) or any social factors that may inhibit uptake of colposcopy would allow improvements to the service to be identified.

Little is known from the patient perspective regarding preferences for the delivery of colposcopy services. The literature that does exist has either been generated in the US, where access to services may be influenced by women's financial situation, or prior to the introduction of nurse colposcopists. Studies have been carried out investigating women's anxieties regarding cervical screening and colposcopy, but few have investigated their experience of the service as a whole. One early study, undertaken in 1983, found that women experienced considerable emotional upset upon diagnosis of cervical abnormalities, that treatment caused distress, and that it was likely that the sensitivity of the cervix, and stressfulness of the whole procedure, had been underestimated [10]. Recommendations were made at that time for service improvements. Due to changes in the colposcopy service over the last 20 years, the introduction of nurse colposcopists and new techniques, this research now needs updating.

Relatively high rates of non-attendance for appointments (up to 25%) [4] indicate that patient satisfaction may not be being fully addressed with consequent inefficiencies in service delivery. There is a need to investigate the experience women now have of colposcopy appointments in the UK, given the changes to service delivery, high default rates [4,9] and the potential psychological and emotional cost to women [11,12]. Investigation of patient experience and preferences provides valuable information that can be fed back to service providers in order to improve the service and ultimately improve patient satisfaction and attendance. In addition to determining the effectiveness of the service offered, it is necessary to establish the appropriateness of delivery from the user perspective; this can only be achieved by more fully understanding women's experiences. This study therefore aims to investigate women's experiences of colposcopy, their preferences and any preconceptions they may have.

The principal research aims are to: 1. Gain an insight into the experience that patients have of the colposcopy service, 2. Identify patient preferences for appointments with the colposcopy service, 3. Make suggestions for service improvements.

## Methods/Design

This study has been designed as a two stage, mixed method project. Stage one will involve in-depth interviews with new colposcopy patients to ascertain their experience of colposcopy services. This qualitative stage will generate factors thought to be important by service users in their experience. Stage two will utilise a choice based quantitative technique to identify women's preferences and determine the representativeness of factors generated through the interviews.

### Stage one – Patient interviews

The study will use qualitative in-depth interviews to investigate women's opinions. This approach has been chosen as the data collection method of choice for this study for two main reasons: 1) Due to the fact that current knowledge is limited in this area, this technique allows hypotheses to be generated rather than enforced by the researchers own opinions; 2) One-to-one interviews are recognised as the best way to gather data on potentially sensitive subjects [13].

#### Recruitment of interview participants

The sample of women will be identified from those invited to attend Birmingham Women's or City Hospital for a colposcopy appointment arising from routine screening (between February and May 2006). Purposive sampling [14] will be employed to select a range of individuals who vary in age, socio-economic background, ethnicity and referral reason.

**Figure 1:**
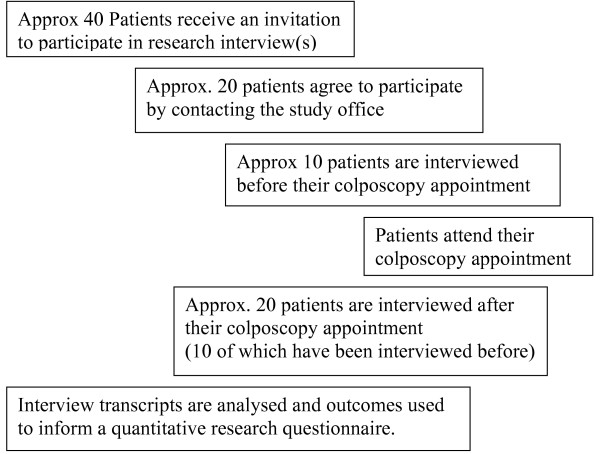
Interview flow chart.

Recruitment will continue until theme saturation occurs. At present that number is unknown, so it is proposed that 30 interviews will be conducted, involving 20 patients, illustrated in Figure 1, to limit the chance of missing any important issues [15,16]. In order to assess women's preconceptions regarding colposcopy half of women interviewed (n = 10) will be interviewed before their colposcopy appointment, then again later, after their appointment. The other half (n = 10) will be interviewed only after their appointments. The distribution of before and after appointment interviews has been made to balance the need to ensure that clear and accurate data are collected but the least undue anxiety is caused to patients. To elaborate, it is difficult to remember accurately what preconceptions people may have held before an event once it has taken place, so some interviews need to occur before the event. However, it will not necessarily be the case that preconceptions change, therefore in parallel some patients will be interviewed only after their appointment. If it becomes apparent that there is no difference between the two groups in terms of changes in preconceptions, no further interviews will take place before the appointments, only after, to reduce unnecessary anxiety i.e. women are expected to be most anxious prior to the colposcopy procedure.

#### Invitation method

An invitation letter will be posted to patients with their colposcopy clinic appointment letter. Potential participants will be recruited on an opt-in basis. The individuals interested in taking part in the research will be able to contact the study office to gain more information about the study and arrange a convenient place and time for interview by using the telephone number given on the invitation letter. Alternatively, they will have the option of posting a reply slip to the study office advising of their interest in taking part and giving details of a phone number they would like to be contacted on. Once personal contact is made by phone any questions about the study will be answered and a convenient time and place for the interview to take place will be arranged.

#### Patient interviews

Consent will be taken by the same researcher (DS) for every participant. The participant will have already received a patient information sheet by post and therefore had time to consider participation. At the time of arrival for the interview, the participant will be reminded of the purpose of the research, their rights to withdraw and given an opportunity to ask questions. The researcher will then go through the consent form with the participant which will act as documentary evidence of the consent process. Copies will be given to the patient and stored in the patient's hospital notes and the research file. Where a second interview is to be conducted it will be discussed with the participant at the end of the first interview.

Those individuals unable or unwilling to give informed consent will be excluded. Participants who may not understand verbal explanations will only be included where their comprehension is such that informed consent can be guaranteed, otherwise they will be excluded. Where language is a barrier, hospital interpreters could be used, however this is only likely where the patient is particularly keen to participate, given the sensitive nature of the subject. It is recognised that a balance exists between not excluding population sub-groups as research participants and embarrassment that may occur by the use of a translator. It is also recognised that where sensitive subjects are discussed participants may choose to omit information if a third party is present.

The interviews will be tape recorded (with the participants' permission) and transcribed by the researcher. All data will be collected by a single researcher to ensure consistency is maintained.

#### Development of interview schedule

A topic guide for the interviews has been developed based upon information gained from existing literature. This interview schedule has been piloted within the Department of Primary Care & General Practice at the University of Birmingham on women in the appropriate age group who have, and have not, undergone colposcopies (n = 6). The schedule has been adjusted according to their comments.

#### Analysis of qualitative data

Once interview data has been collected a thematic framework will be developed under the guidance of the qualitative research supervisor. Analysis of the interview data will be using the Framework model developed by the National Centre for Social Research. Using Framework analysis will involve familiarisation with the data, followed by; identification of recurring and important themes, indexing, charting, data abstraction and finally, investigation and interpretation.

Reasons underlying women's preferences for aspects of the service will be investigated. Relevant related issues, such as the influence of the individual colposcopist on perceptions of care, and the importance of the health professional-patient relationship are issues of particular interest and potentially modifiable factors in delivery of the service, these will be looked for in the data. Perceived barriers to accessing the service will be investigated, particularly in relation to different patient types.

#### Outcome measures of Stage one – Patient interviews

The outcome of the analysis will comprise the main factors believed by patients to be important in their experience of the colposcopy service. These factors will inform the design of a larger quantitative questionnaire based study utilising discrete choice analysis (also known as discrete choice experiment). This second stage will enable access to a larger, more representative population and the provision of generalisable data.

### Stage two – Discrete choice questionnaire

This part of the project will consist of administering a discrete choice questionnaire to women who have previously attended a colposcopy appointment, asking about their experience of colposcopy. It will be a quantitative investigation aiming to determine the representativeness of qualitative findings and to produce generalisable data. This discrete choice methodology of eliciting user preferences employs various hypothetical scenarios, which incorporate different combinations of factors, to gain a measure of the relative importance of issues to women. The factors included will have been identified through in-depth interviews, in stage one of this project, as important in women's experience of colposcopy.

#### Discrete choice methodology

The technique of discrete choice experiment (which has also been called conjoint analysis) was developed during the 1990s to elicit patients' views on healthcare [17]. The technique has evolved over the last thirty years in disciplines as varied as econometrics, transportation, marketing, decision statistics and biostatistics, where it is also known as stated choice methods [18].

The basic properties of the technique involve identification of consumer preferences for healthcare by examining the trade-offs that individuals make between differing aspects of their healthcare [19]. Each individual is offered a choice of scenarios and asked to state which scenario they would prefer. This allows ranking of characteristics of a service and consumer preferences to be deduced. Behavioural responses to changing opportunities may then be predicted in order to optimise a service.

#### Best-worst scaling technique

A recent adaptation to the discrete choice experiment is best-worst scaling. This technique captures additional information by asking respondents to perform a different task to that required by the tradition discrete choice experiment [20], eliciting what they find best and what they find worst about a scenario, whereas traditional discrete choice experiment asks them to choose which scenario (in total) they prefer, giving no additional opportunity to express preferences within the scenario. They also allow the respondent to reject both scenarios.

Flynn at al (2006) highlight the advantage of best-worst scaling over traditional discrete choice experiments as providing additional insights for health service researchers and allowing the impacts of attributes to be compared. They also propose that a best-worst scaling exercise is potentially easier for respondents, thus providing an additional benefit over the use of traditional discrete choice experiments [20].

Given the potential advantages of the best-worst scaling form of discrete choice experiment in terms of both additional outcome data and ease of use for respondents, this format was chosen for the design of the quantitative questionnaire.

#### Recruitment of participants

The sample of women will be drawn from those invited to attend Birmingham Women's or City Hospital for a follow-up appointment within the colposcopy department. Two methods of recruitment will be employed; i) a poster for those who attend the clinic appointment and ii) postal, for those who do not attend their appointment. Recruitment will be conducted at consecutive follow-up clinics during October and November 2007.

The questionnaire technique proposed is a discrete choice experiment using best/worst scaling. This methodology is new and has been applied in the healthcare setting to elicit dermatology patients' preferences for appointments [21] and older people's views on their future care (Unpublished work by J Coast 2007). An accepted method for calculating power is unavailable because this is a relatively new type of discrete choice experiment utilising a different choice procedure on the part of respondents.

Using the published studies as examples for sample size, the most recent published study achieved significant results with 30 individuals per subgroup of interest [21]. The sample size will therefore be based upon the need to gain a minimum of 30 individuals per subgroup of interest. Two subgroup sets are suggested from phase 1 of this study; i) Based upon treatment received and ii) Based upon which hospital they attended. Other subgroups may be present but not immediately apparent, therefore the sample size will aim to achieve 100 completed questionnaires, including a minimum of 30 patients who have received treatment, 30 patients who have not received treatment and 30 patients from each hospital site.

#### Invitation method

Initially, patients will be invited to take part in the research by means of a poster displayed in the waiting area for colposcopy patients. The poster will explain the reason for the research and what is required of participants. The colposcopy clinic staff will be fully briefed and will have patient information sheets and blank questionnaires to give to women who ask for them. The researcher will be present during follow-up clinics to assist women with completion of the questionnaire.

Women will be asked to complete the questionnaire while they wait, if they have time. If they do not have time they will be provided with a reply-paid envelope to post the completed questionnaire back to the University at their convenience. In addition women will be provided with a withdrawal form and pre-paid envelope, so that if with hindsight they decide not to participate in the study return of the slip will result in withdrawal of their data from the study and destruction of their questionnaire.

Women who do not attend their colposcopy appointment will be posted information about the research study together with a questionnaire, unless they are identified by a consultant as excluded (e.g. in the case of invasive disease diagnosis). One reminder letter will be sent to them about the study. No further contact will be made.

A formal consent form will not be used for this questionnaire survey. Completion of the questionnaire will be taken to imply consent. Women sent a questionnaire would be invited to return it blank in the pre-paid envelope if they wish to decline participation. All questionnaires distributed will be accompanied by a patient information leaflet.

#### Questionnaire design

The main part of the questionnaire will be the best-worst scaled discrete choice experiment, but in addition other information will be collected from the participants about themselves and their prior experience of colposcopy to enable the role of socio-demographic and treatment factors to be established.

Participant's age will be recorded to ensure that respondents' age range represents that of colposcopy service users. Census based classification of ethnic groups was chosen at the coarse classification of level 1 giving general information about ethnic group. Although it is preferable to have more detail, given the relatively small numbers of participants it would be inappropriate to use the more detailed level 2. Collection of ethnic group information allows comparison to census data to determine the representativeness of responders.

Literature suggests that some groups of people have more difficulty accessing the service e.g. those with childcare commitments. Therefore, occupation and number of children may impact upon service preferences and are included in the questions.

In order to make sure that important factors regarding the colposcopy service have not been overlooked by the interview data and thus omitted from the choices in the questionnaire, an open field is offered for respondents to make any additional comments upon what they feel are important factors.

#### Development of questionnaire

**Figure 2:**
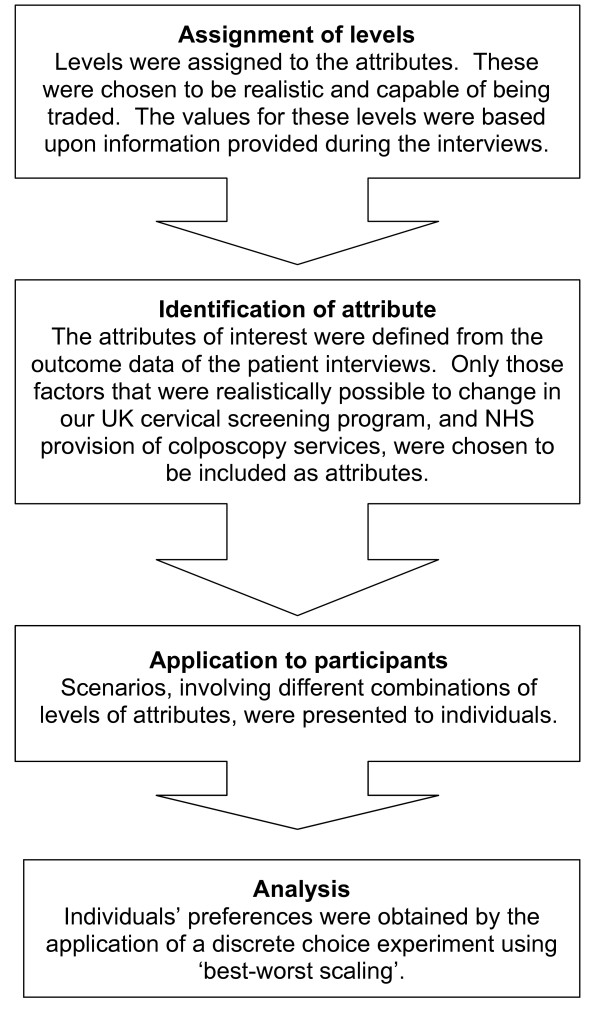
Stages of choice development (modified from Ryan [19]).

The individual attributes or characteristics required for the best-worst scaling discrete choice experiment will be developed through the recognised stages [19] in Figure 2.

**Figure 3:**
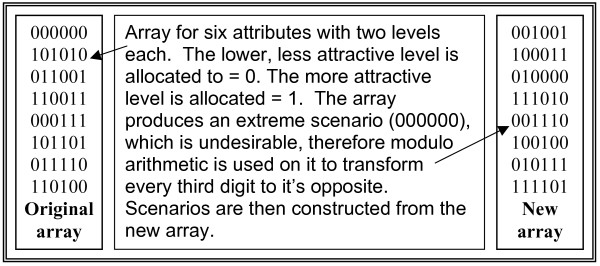
Orthogonal array and it's transformed version.

Six attributes with two levels each were identified from the interviews, which gave a full factorial design of 64 scenarios. It would be unrealistic to expect participants to complete a questionnaire of this length and this number of scenarios, therefore a more pragmatic approach uses a fractional factorial design. This fractional design is achieved with the use of an orthogonal matrix that ensures that with a smaller number of scenarios each combination of factors occurs an equal number of times. The orthogonal array is presented in Figure 3. This reduces the number of scenarios required for the experiment to eight and offers a robust design for the discrete choice experiment [22].

The orthogonal array was sourced from N.J.A. Sloane's library of orthogonal arrays [23]. It was chosen to represent six characteristics with two levels each. The array contains an extreme scenario, where all the characteristics have their most favourable or least favourable option present. This extreme is undesirable in the questionnaire because it causes the non-completion rate of the questionnaire to increase; this happens because where all options are optimised or minimised people find it difficult to choose the worst of all good choices or the best of all bad choices. Only fully completed questionnaires will be analysed so this difficulty associated with completion would impact upon analysis. Therefore, to overcome the non-completion issue the matrix can be transformed using modulo arithmetic to a form without extreme scenarios. This transformation changes every third digit giving a new array where no extreme scenarios are present.

Scenarios are constructed using the matrix to determine the choice of characteristic level for each scenario. Once the orthogonal array was selected and applied to the characteristics and their levels in order to construct the questionnaire, randomisation of order was applied. Within each scenario the order in which the attributes occurred were randomised. In addition, the order in which each appointment scenario appeared in the questionnaire was randomised.

This randomisation achieved two outcomes. Firstly it ensured that participants would be less likely to exhibit a bias in answering the first or last characteristic repeatedly, because they may remember it more easily than those in the middle. Secondly, any systematic pattern in the orthogonal array was not replicated in the order of scenarios and characteristics by this fully randomised questionnaire design.

Six main factors were found, during Stage one, to influence women's experience, these included:

• The attitude of staff.

• Delays and waiting time until referral.

• Viewing the monitor present in the consultation room.

• The provision of information before their appointment.

• The gender of the colposcopist.

• The feeling of being rushed whilst in their appointment.

These were broad issues and incorporated a range of specific points that women raised during the interviews. Realistic levels (representative of real life) were assigned to these characteristics in order to construct the questionnaire.

#### Piloting of questionnaire

The questionnaire has been piloted on women (n = 11) within the cervical screening age group (25–64 years old), a number of whom had attended colposcopy appointments in the past. Comments made in relation to ease of understanding, use of wording and presentation led to revision of the questionnaire. The average length of time taken to complete the questionnaire was 12 minutes.

#### Analysis of quantitative data

The analysis will use a weighted least squares method to regress the choice of best-worst pairs as one data point against the attribute level least preferred. The accept/reject 'would you attend this appointment' question will be analysed using a random effects logit model to adjust for clustering in individuals' responses [21].

### Ethical approval

Ethical and hospital R&D approval were applied for in two stages. Initially applications were made in January 2006 to South Birmingham Local Research Ethics Committee (LREC) and the R&D departments for both hospital trusts to conduct patient interviews. These were granted in March 2006, REC reference number 06/Q2707/13, R&D approval number 10030602 project reference SWA003. Subsequently, application was made in May 2007 to Sandwell & West Birmingham LREC and the R&D departments for both hospitals to administer the questionnaire. These were granted in September 2007, REC reference number 07/Q2709/61, R&D approval number 01100701.

## Discussion

Colposcopy is a common procedure and one that is associated with raised anxiety among women experiencing the service. Little is known about women's experience of the service or their preferences for service delivery.

The outcomes of the study will comprise a description of women's experience of colposcopy and establishing their preferences for how aspects of the service should be provided. Women's preferences will be fed back to service providers to enable improvements to the service to be made.

## Abbreviations

UK = United Kingdom

US = United States

BSCCP = British Society for Colposcopy and Cervical Pathology

LREC = Local Research Ethics Committee

## Competing interests

The author(s) declare that they have no competing interests.

## Authors' contributions

All three authors contributed to development of ideas and design of the study. DS wrote the first draft of the manuscript, which has been commented on by the other authors. All authors read and approved the final manuscript.

## Pre-publication history

The pre-publication history for this paper can be accessed here:


